# Surgery for interplanetary space missions

**DOI:** 10.1093/bjs/znag005

**Published:** 2026-03-13

**Authors:** Raghav Khanna, Yang Li, Matthew Cook, Preeti Sawant, Raymond Hounon, Danielle Carroll, Lakita Lowe, Lukas Lindenroth, Toktam Mahmoodi, Nicholas Raison, Alejandro Granados, Anu Ojha, Christos Bergeles, Alberto Breda, Sebastien Ourselin, Prokar Dasgupta

**Affiliations:** Guy’s, King’s and St Thomas’ School of Medicine, King’s College London, London, UK; School of Biomedical Engineering and Imaging Sciences, King’s College London, London, UK; School of Biomedical Engineering and Imaging Sciences, King’s College London, London, UK; UK Space Agency, Didcot, UK; National Board of Examinations in Medical Sciences, New Delhi, India; Alibaba Cloud Intelligence Group, Hangzhou, China; UC Space Health, University of California San Francisco (UCSF), San Francisco, California, USA; NASA Johnson Space Center, Houston, Texas, USA; School of Biomedical Engineering and Imaging Sciences, King’s College London, London, UK; Centre for Telecommunications Research, King’s College London, London, UK; Department of Urology, King’s College Hospital NHS Foundation Trust, London, UK; School of Biomedical Engineering and Imaging Sciences, King’s College London, London, UK; UK Space Agency, Didcot, UK; School of Physics and Astronomy, University of Leicester, Leicester, UK; School of Biomedical Engineering and Imaging Sciences, King’s College London, London, UK; Department of Urology, Fundació Puigvert, Barcelona, Spain; School of Biomedical Engineering and Imaging Sciences, King’s College London, London, UK; Department of Urology, Guy’s and St Thomas’ NHS Foundation Trust, London, UK

## Abstract

As human spaceflight expands beyond low Earth orbit, the ability to deliver advanced surgical care in space becomes critical. Current medical provisions on board the International Space Station (ISS) are geared towards treating low-risk conditions, with a ‘stabilize-and-evacuate’ principle for more complex cases—an approach that is not viable for extended missions to the Moon and Mars. This review summarizes research conducted around space surgery, with a particular focus on surgical robotics. Experiments in parabolic flight and analogue environments demonstrate that, provided the operator, patient, and instruments are restrained, surgical skill is largely unaffected by reduced gravity. Robotic surgery has primarily been explored in remote undersea habitats and in limited flight studies. There are several challenges to the implementation of surgical systems in space, including size, weight, and power constraints, communication latency, and crew training. Means of fluid and debris containment, provision of anaesthesia, and postoperative recovery in altered physiology must also be considered. The key features of an ideal space surgery robotic set-up are outlined. It should be compact, multifunctional, adaptable, reliable, and optimized in technical design and material composition for use in habitable volumes. Such systems should incorporate artificial intelligence (AI)-driven decision-making support, variable autonomy, and human-in-the-loop control. Crew members must be trained and supported to deliver and recover from surgical care in space. Cloud and edge computing will mitigate latency while expanding on-board data processing capabilities. Although not yet operationally mature, robotic surgery is a critical capability for future exploratory space missions, but requires continued multidisciplinary development.

## Teaser

This review summarizes previous work on surgery in microgravity and proposes robotic surgery as the best solution for positive astronaut-care outcomes, with a complete technical workflow that covers the crew, robot, and environment.

## Introduction

Lewis and Clark’s expedition through the Western USA in the early 19th century is a tale of bold curiosity and determination. All but one member of the group returned safely back to St Louis after nearly 2.5 years^1^. Sergeant Charles Floyd, quartermaster of the expedition, died about 3 months into the expedition of what was then described as biliary colic. Historians today agree that he died of acute appendicitis and peritonitis^1^. Treating him was impossible, as the group only carried basic medical supplies and was far away from any settlements or physicians. In any case, Floyd’s death was unavoidable, as there were no medical or surgical treatments for appendicitis in 1804.

Medicine has evolved considerably since then, while the thirst of humans to explore remains unchanged. Space has once again become the next frontier; humans are poised to return to the lunar surface and are preparing the first planetary exploration to Mars. In doing so, humans will be far beyond the safety of Earth-based care^2^. Astronauts will travel farther, stay in space for longer, and encounter even harsher, more unpredictable environments from where returning home in a medical emergency is impossible^2^.

Until now, the availability of rapid evacuation has largely rendered on-site surgical capabilities unnecessary. This reliance on rapid evacuation is evident in contemporary spaceflight: in early 2026, the National Aeronautics and Space Administration (NASA) conducted the first reported medical evacuation from the International Space Station (ISS)^3^. Although details of the medical concern were not publicly disclosed, the event was sufficiently serious to prompt the cancellation of a scheduled spacewalk and the early termination of Crew-11’s mission. The ISS continuously maintains re-entry capability for its full crew to enable urgent return to Earth; in this instance, a SpaceX Crew Dragon spacecraft was used to evacuate the four members of Crew-11.

Sergeant Floyd’s death serves as a historical lesson for modern exploration. To prevent treatable conditions from becoming fatal in space, space missions must have the ability to provide more advanced medical and surgical care without Earth-bound support. Surgical robotics now appears to be emerging as a mission-critical feature, providing diagnostic and decision-making support, undertaking semi-autonomous procedures, and relaying medical data back to mission control^2,4^. Surgical robotics has the potential to compensate for the highly constrained environment inherent to space exploration.

## History of medical risks and capabilities in space

**Fig. 1.**
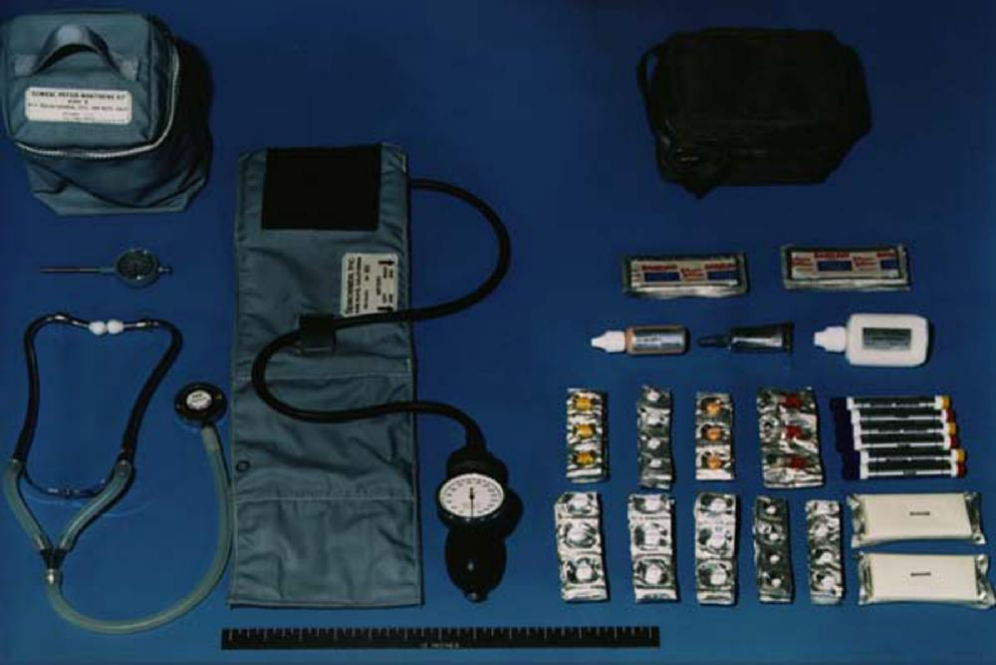
Medical kit carried on the Apollo missions. Image credit: NASA.

The first human spaceflight programme of the USA, Project Mercury, began in 1958, with the longest Mercury flight spending nearly 35 h in orbit. Astronauts only carried antiemetics, analgesics, and stimulants^5^. The next missions, part of Project Gemini, proved that multiday missions in space were survivable, with astronauts also carrying decongestants, antibiotics, and eye drops^5^. For subsequent Apollo missions, the on-board pharmacy added medications for arrythmia, eye, ear, nose, and throat pathologies, antihistamines, broad-spectrum antibiotics, analgesics, and gastrointestinal medication (*Fig. 1*)^4^. Finally, Skylab and the Space Shuttle programme introduced equipment for medical procedures to space, including equipment for intravenous cannulation, airway management, wound closure, and dental care^5,6^. All projects in the early crewed spaceflight era continuously monitored the vital signs of astronauts, including ECG, respiratory rate, blood pressure, and temperature^7^.

Space Station Freedom (SSF) was a planned, crewed space station announced in 1981 that eventually merged with the Russian Mir-2 concept to become the ISS. While several concepts from SSF were transferred to the ISS, one key feature was left out—a comprehensive health maintenance facility (HMF), capable of preventative, diagnostic, and therapeutic medical and surgical care^8^. This had been planned to include a waist-level operating table (eventually replaced by a crew medical restraint system (CMRS)), theatre lighting, diathermy, suction, and a full anaesthetic suite^8^. Ultimately, the inclusion of an assured return vehicle meant the HMF never materialized^8^.

**Fig. 2.**
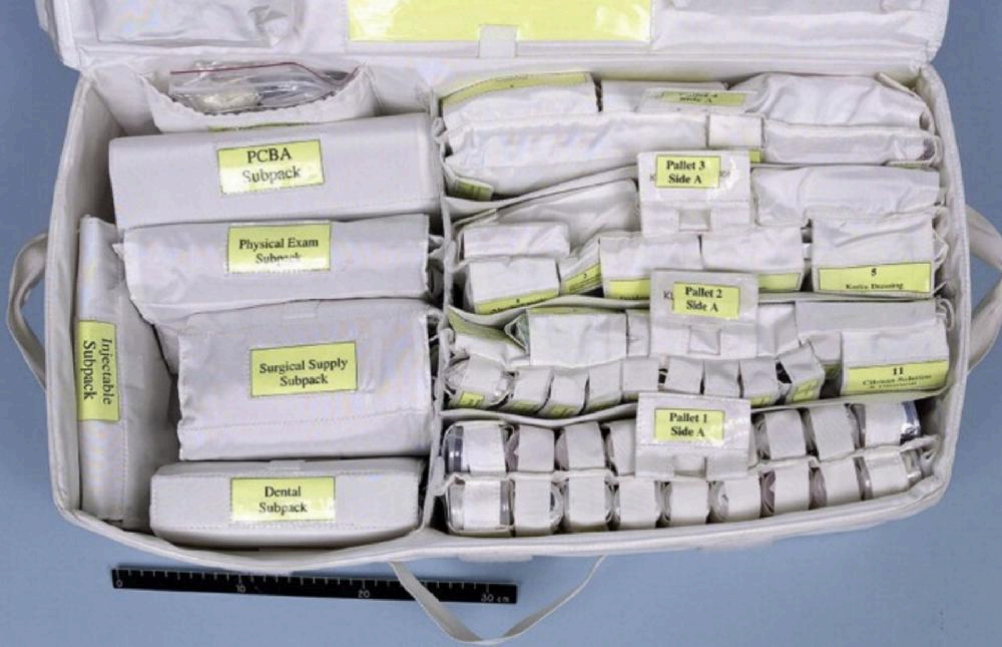
Ambulatory medical pack on the ISS Note the surgical supply subpack on the left. ISS, International Space Station; PCBA, Portable Clinical Blood Analyzer.

Today, medical care capabilities on the ISS include advanced trauma life support (ATLS) and advanced cardiac life support (ACLS), basic surgical instruments, sutures and local anaesthetic, ultrasonographic machines, intravenous access kits, dental tools, advanced airway management kits, variable flow oxygen, and a range of drugs, including antiemetics, antibiotics, steroids, etc. (*Fig. 2*)^9^. Crew medical officers (CMOs) receive 40 h of preflight training and can measure vital signs, conduct physical exams, diagnose basic conditions, administer medication, appropriately utilise the onboard equipment to deliver routine and emergency care, and work with a crew surgeon in mission control to treat and evacuate patients^10^. Crew surgeons require nearly 400 combined hours of classes, on-the-job training, and console training to qualify for duties^11^ . All crew members also receive ACLS training and attend preflight ‘megacodes’—simulated medical emergencies on the ISS^12^. Of course, the key focus is on prevention. Exercise is regularly scheduled for all astronauts, the crew surgeon makes regular crew assessments, and dieticians track fluid and food intake. Astronaut candidates are carefully screened for any potential pathology that may declare in space, with space agencies now increasingly focused on medical issues that will impact missions >3 months. While research has explored the use of prophylactic surgery (for example appendicectomy and cholecystectomy), evidence does not support this practice and to date such an approach has not been implemented^13^.

Despite stringent measures for prevention and extensive prescreening, medical events are unavoidable, as highlighted by the recent ISS medical evacuation. In another case, a venous thromboembolism of the left internal jugular vein was discovered in an astronaut on board the ISS^14^. After multispecialty discussions, enoxaparin available on the ISS was prescribed until apixaban and reversal agents were delivered on a resupply mission.

Quantifying the risk of surgical incidents enables the need-based development of technologies necessary to facilitate space surgery. It also enables mission planners to create a human system risk board that minimizes the risk of and accounts for medical and surgical emergencies^15^. Based on the risk of emergency events in the general population, the risk for a seven-person crew mission to Mars stands at one event every 2.4 years^16^. NASA maintains an integrated medical model (IMM), a list of 100 medical conditions most likely to occur during space exploration, with their associated single-mission probabilities^17^. This includes 27 conditions that may require surgery, primarily acute abdominal pathologies and traumatic injuries^18^. The IMM has also been used to predict outcomes of these medical conditions on standard and accelerated four-person Mars missions. Simulations determined the likelihood of a situation that would warrant immediate patient evacuation on the ISS to be 29.4% and 6.3% for standard and accelerated missions respectively; the risk of crew death was determined to be 1.58% and 0.57% respectively^19^. However, mortality figures did not account for the fact that immediate crew evacuation during a Mars mission is generally impossible and so the true figures are likely to be higher. For an accelerated mission, the leading surgical drivers for evacuation were kidney stones and dental abscesses while stroke and appendicitis were the main surgical contributors to loss of life. For the standard-duration missions, traumatic haemorrhage is seen as an additional main risk to life.

## Open surgery in microgravity

Open surgery is well suited to emergencies, as well as low-resource and extreme-isolation settings, as it does not require specialized tools, advanced visualization, or cavity expansion.

The first space surgical experiments included performing laparotomies on rabbits while in parabolic flight^20^. From the 1980s onwards, basic surgical skills, haemorrhage control, laparotomies, ACLS techniques, and fluid-containment strategies were evaluated using phantoms and models. Studies identified two baseline requirements for an open surgical set-up in space: a containment unit to prevent cabin contamination by surgical fluids and a restraint system to secure the patient, operator, and surgical instruments^20^. Surgical skill does not appear to be impaired in microgravity. With the surgeon comfortably restrained to the patient, performing simple suturing in microgravity or lunar gravity appears no more difficult than suturing in standard gravity^21,22^. While task completion times were slower in reduced gravity, suture placement and suture lines showed near equivalence to the results achieved in 1 g. Another study evaluated the performance of ten surgeons in performing damage-control surgery (laparotomy) followed by the identification and packing of an unknown haemorrhage site using a high-fidelity torso simulator^23^. While 0 g was subjectively considered harder overall, most phases of the operation were rated equivalent to 1 g. Indeed, the opening of the abdominal wall was quicker and there was reduced blood loss during bleeding-site identification in 0 g^23^.

Strategies for containment of surgical fluids in 0 g have been extensively explored. The life sciences glovebox and microgravity science glovebox on board the ISS are containment units that use dual barriers, continuous negative pressure, multilayer filters, and charcoal filter stacks. These are coupled with crew-access glove ports to completely isolate the internal chamber from the habitable environment^24^. Both are rigid units that conform to the international standard payload rack (ISPR) architecture. It is recognized that, in practice, a surgical containment unit would need to cover any volume of the patient (for example entire limbs or a section of the abdomen, chest, or head). In a series of surgical procedures, including carotid artery repair, laparotomy, and abdominal aorta repair on rabbits in parabolic flight, fluid containment was achieved with a surgical overhead canopy (SOC)^25^, a laminar flow device (LFD), suction, and swabs. Within the SOC, the surgical tray was attached to the LFD and was built to secure ferrous instruments (through a magnetic section) and sharps (using a Styrofoam block). The SOC also had pockets for the disposal of waste, procedure packs containing sterile instruments and other supplies, and a pair of arm ports with sleeves on both sides for the operators. Results of use in microgravity demonstrated successful containment of all fluids and completion of surgical tasks, with equivalent outcomes compared with 1 g^25^. Indeed, studies involving fluids and blood have observed that fluid management is not as challenging as it may appear. In microgravity, surface tension dominates and blood tends to form domes and adhere to the abdominal wall and folds in the anatomy^25^. High-pressure arterial blood can form a stream of droplets; however, the flow of blood is generally disrupted at the wound site and forms domes similar to venous blood^25^.

ATLS procedures should be within the capabilities of astronauts and spacecraft. Chest-tube insertions, artificial ventilation, intravenous infusions, Foley drainage, wound closure, tracheostomy, and peritoneal lavage on porcine models have been tested in parabolic flight^26^. Most procedures were completed without difficulty compared with 1-g conditions. Chest tubes were placed under real-time telementoring by a ground-based surgeon and a closed, one-way drainage circuit was implemented that prevented back-flow even when the patient’s orientation was changed. However, percutaneous peritoneal lavage was deemed unsafe due to the bowel rising anteriorly, creating a significant perforation risk^26^.

The NEUROLAB/STS-90 mission conducted on board the space shuttle Columbia involved the only surgical procedures performed in space^27,28^. During NEUROLAB, tail-vein cannulations, thoracotomies, laparotomies, craniotomies, anaesthesia, and wound closure were performed on mice within a sealed general-purpose workstation. Dexterity, fine motor skills, and instrument manipulation were not significantly altered in 0 g^27,28^. It should be noted the NEUROLAB payload specialists were medically qualified (one physician and one veterinarian) who underwent focused training in microgravity conditions for nearly 2 years to develop and refine the surgical skills used during the mission.

## Non-robotic minimally invasive surgery (MIS) in microgravity

In microgravity, anatomical cavities act as their own containment units^20^. Non-robotic MIS has been extensively evaluated in microgravity conditions. A study of laparoscopic visualization and excision of gynaecological adnexa in porcine models found that visualization was unaffected by microgravity^29,30^. While it was hypothesized that floating bowel and fluids would pose an issue, the bowel was pulled away from the abdominal wall due to the elastic mesentery and the cavity itself changed shape from oval to round, increasing the anterior volume. Fluids did not diminish the view, as surface tension dominates, and blood remained adherent to the abdominal wall during surgery. It was noted, however, that blood did impair visualization when it formed sheet-like layers around organs. However, thoracic surgery was challenging: visualization was impaired by anteriorly displaced lungs that could not be deflated through selective intubation due to the complexity of the procedure.

Completion of four basic tasks by 20 surgeons on a laparoscopic trainer while in parabolic flight was reported in 2004^31^. The number of successful task completions in microgravity decreased compared with 1 g. Panait *et al*.^31^ highlighted that outcomes were promising and could be improved if participants received more preflight familiarization and training on laparoscopic techniques. A subsequent study of hand-assisted laparoscopic appendicectomy in microgravity^32^ reported successful completion, with adequate containment of all equipment and fluids inside the unit. Meanwhile, a further study found laparoscopic surgery in porcine models without insufflation^33^ to be impractical.

In 1999, ultrasound-guided stenting was achieved using a flexible cystoscope in porcine models during parabolic flight^34^. The procedure was performed by a three-member team—a surgical assistant, the surgeon, and an ultrasonographer. Minimal irrigation fluid was used as the saline tended to cling to the instrument and pass upward until it floated away into the cabin. Ultrasonographic visualization was adequate and allowed for effective stent placement. Images collected using ultrasonography and cystoscopy in microgravity had no significant differences compared with those collected in 1 g. While the procedure was completed successfully, Jones *et al*.^34^ did highlight limitations of their setup due to the lack of fluoroscopy. It is also recognized that short 20–25 s intervals of microgravity during parabolic flight may not accurately reflect results in a continuously weightless environment.

Among the proposed containment systems for space surgery, the aqueous immersion surgical system (AISS) currently represents the most mature design^35^. The AISS is a rigid, clear dome that can be placed over skin for complete filtration of blood, insufflation gas, debris, and diathermy smoke. The filtration is achieved using saline that is circulated in the dome using a dynamic, pressure-adjustable fluid-management system. Saline pressure can be increased to stem active bleeding. The AISS can be used for open surgery or MIS, with no-leak ports for laparoscopic instruments. An allied multifunction surgical device (MFSD) provides irrigation, suction, and illumination through a three-dimensional (3D)-printable, push-button handheld interface^36^, with future iterations expected to add visualization and cautery. The AISS and MFSD have been tested on board the Virgin Galactic Unity spacecraft in suborbital flight, demonstrating the integrated system’s ability to maintain stable dome pressures, clear mock haemorrhages, and continuously exchange fluid in various flight profiles^37^.

## Robotic surgery in microgravity

The first experiments examining the use of surgical robots in space were performed during NASA extreme environment mission operation (NEEMO) 7, NEEMO 9, and NEEMO 12^2,38^. NEEMO 7, 9, and 12 were part of an undersea space-analogue programme based out of the Aquarius marine laboratory, 60 feet below sea level^38^. NEEMO 7 saw the deployment of the AESOP robot (Computer Motion, Inc., Goleta, CA, USA)^2,39^. The ‘aquanauts’ were telementored by surgeons in Canada to perform five key tasks, including ultrasound-guided imaging and abscess drainage, vascular repair, cystoscopy and stone removal, and laparoscopic cholecystectomy on a simulated cadaver. Successful deployment of ultrasonography, basic surgical instruments, flexible cystoscopes, laparoscopic instruments, and the AESOP robot was reported^39^, with successful telementoring to facilitate complex surgical tasks. It emphasized the need for a robust telecommunication network, mentor familiarity with robotic systems, and the adoption of standardized terminology for effective communication^39^.

The next mission, NEEMO 9, saw the deployment of a full-scope MIS robot, the M7 (SRI International, Menlo Park, CA, USA). Aquanauts performed external fixation of a tibial fracture, knee ultrasonography and arthroscopy, haptic manipulation of virtual tissue, and assembly of the M7 for telesurgical suturing^39^ . All tasks were performed successfully through telementoring in 750-ms signal latency and 3-s delay mimicing Earth-Moon communication^2^. Although suturing was successful, it took 10 minutes to tie a single knot under lunar latency.

Given potential latency issues, NEEMO 12 explored the semi-autonomous use of the M7^40^. The M7 used an adaptable tool interface to mount an ultrasonographic probe and needle. The ultrasonographic probe was guided by a surgeon under a latency of 500–1000 ms to visualize a simulated vessel. After confirming a satisfactory view, the surgeon selected the target using ultrasonography and the needle was autonomously inserted along its pre-positioned axis^40^. A second surgical robot, the RAVEN (UW BioRobotics Laboratory, Seattle, WA, USA), was also teleoperated to perform basic MIS tasks and manipulation of simulated Moon rocks^40^.

The M7 robot was further evaluated in parabolic flight to test the effectiveness of acceleration compensation in variable g conditions^41^. The team used a three-axis inertial measurement unit to calculate acceleration and g levels, and introduce a compensating damping to the leader manipulators. Operators reported that acceleration compensation greatly improved the usability of the robot in placing a running suture while in variable g^41^. However, compensation did not improve usability in fixed 0-g conditions.

In 2024, the MIRA surgical robot (Virtual Incision, Lincoln, NE, USA) was sent to the ISS and used to perform a series of basic tasks in teleoperated and autonomous modes^42,43^. This marked the first instance of a surgical robot deployed in space. ‘SpaceMIRA’ was shortened to fit into an EXPRESS rack^42^ and first used to perform autonomous cutting of rubber bands. It was then teleoperated under a latency of 600–800 ms to perform manipulation and cutting tasks^43^. Formal results analysing the performance of the robot in microgravity and comparing it with the performance of the robot in 1 g are awaited.

Apart from dedicated robotic MIS systems, research has explored whether space robots can function as surgical assistants^44^. Robonaut 2 is a humanoid robot introduced to the ISS in 2011 for tasks requiring human-like dexterity. The robot can perform general caretaking tasks under supervised autonomy and, on return to Earth in 2018, has been used to perform ultrasonographic imaging for jugular vein central line placement and laryngoscopy for intubation while being controlled by a surgeon wearing virtual reality (VR) goggles and haptic gloves^27,45^. Limitations remain the size of its hands, lack of support at the thumb base, and lack of complete operator control over wrist and arm motion^45^. Apart from the Robonaut, free-flying devices on board the ISS such as the Astrobee and CIMON are being used for assistance with experiments, payload transfer, and station monitoring^46^. A potential expanded function may be to provide ‘over-the-shoulder’ decision-making support and instrument transport during surgery.

See *Table 1* for the suitability of the three main surgical techniques for surgery in space.

**Table 1:** Comparison of the suitability of the three main surgical techniques for space surgery

Feature	Open	Non-robotic MIS	Robotic MIS
Containment	Required and challenging to implement; rigid containment structures cannot adapt to any surgical site.	Smaller incisions and ports naturally contain, but ports will need to completely seal to prevent gas leakage.	Largely similar to non-robotic MIS. Robotic single-port techniques may reduce the number of incisions needed.
Performance	Depends solely on operator skill, likely no harder than surgery on Earth. Assistive technology restricted to telementoring and decision-making support.	Largely similar to open surgery, but potentially riskier due to floating bowel and reduced visualization.	Can integrate compensatory features and autonomous procedural assistance.
Technical complexity/SWaP	Core-instrument set-up is minimal, although more complex surgery may require on-board fabrication of new tools.	Moderate footprint, requires dedicated devices for visualization, insufflation, etc.	Highest—robot may be bulky, will have a significant power draw, and procedure depends on an absence of mechanical issues.
Patient implications	Large incision; longer recovery, more complications, blood loss, etc. Domino effect on crew availability and resource depletion.	Smaller incision; quicker recovery, reduced complications and blood loss.	Same as non-robotic MIS.
Use case	All forms of surgery, from hard-tissue bone fixation to wound repair.	Soft-tissue MIS only, for instance appendectomy.	Primarily soft-tissue MIS, but could be adapted to soft-tissue open surgery.
Implications for training	Basic surgical skill already forms part of training for CMOs. Will require additional training for more advanced procedures, but this can be done in transit or on a case-by-case basis.	Extensive: specialized skill that is very challenging to acquire. Will require very significant commitment and the use case is limited to soft-tissue MIS.	Technically demanding, but basic proficiency achieved quicker than with MIS. Ideally robot is multiuse, so training is efficient, and skills are maintained over the course of a mission.
Evidence	Extensive: techniques and skills evaluated in parabolic flight and in space, on animal models and phantoms.	Extensive: techniques and skills evaluated in parabolic flight, on animal models and phantoms.	Little evidence. Limited tests in space-analogue environments and a recent evaluation in space, but for simple manipulation.

MIS, minimally invasive surgery; SWaP, size, weight, and power; CMOs, crew medical officers.

## Barriers to implementation of robotic surgery in space

Two core requirements for implementation of robotic surgery in space are recognized: restraining the patient, operator, and instruments, and preventing any fluids and debris from contaminating the cabin environment. However, other challenges must be overcome before space surgery can become a reality.

Any object intended for transportation to and use in space needs to minimize size, weight, and power (SWaP)^44^. Current surgical robots are bulky and heavy, with significant power needs. MIS instruments have a large footprint and it would be difficult to fit multiple robotic arms inside an ISPR. While having a multiuse robot is ideal, research and development even within the same agency can be isolated due to organizational silos, making a universal robot difficult to implement.

Data transfer and latency remain critical limitations. The current distance record for telesurgery stands at 12 000 km—a robotic prostatectomy performed between Morocco and China^47^. Generally, the acceptable one-way latency for telesurgery is 100 ms, with gaps >300 ms considered high risk^48,49^. Communication and data relay between NASA and the ISS is conducted through the tracking and data relay satellite system (TDRSS), with a typical two-way latency of 500 ms^50^. A simple radio link to the lunar surface has a two-way time delay of 2.56 s, while communication with Mars can range from 6 to 40 min based on planetary position^51^. As such, telesurgery beyond the ISS by a ground-based surgeon will remain impossible. Other than latency, the data rate is also limited in the TDRSS, restricting the ability to monitor a procedure with a high level of fidelity. The likely solution to remote operation is a combination of preflight training, robotic autonomy and assistance, and telementoring. However, robotic autonomy has not advanced to a stage where systems can make independent decisions without operator control and such systems are unlikely to achieve this capability in the near future^52^.

Preflight training of astronauts on all potential robotic surgical tasks will prove challenging^53^. All astronauts have primary roles that require extensive training to perform, with CMOs astronauts first and care providers second^53^. The reality of space exploration is that engineering and technical expertise will almost always be perceived as more important compared with acquiring a broad range of medical and surgical competencies^53^. Medical planners might have a long wish list of procedures to include in training, but mission planners are likely to perceive anything but the most essential survival skills as unnecessary^53^. The topic of training also creates several additional questions with regards to who and what to train, what tasks to prioritize, and whether to include contingencies for robotic failure.

In the early days of this new era in space exploration, on-board caregivers will face ethical questions^54^. For example, if a surgical emergency arose on a mission as critical as the first Mars landing, when would the patient’s condition override mission success? Surgical resources will be limited and resource use *versus* retention decisions could arise^54^.

Perioperative physiology and recovery are different in space. Postoperative recovery will be slower in space. Microgravity delays wound healing due to down-regulated production of growth factors, modifications in cell signalling, and reductions in platelet count^55^. Microgravity, radiation, and sleep disruption weaken the immune system^56^. The risk of vascular events rises due to venous stasis, cardiac atrophy, hypovolaemia, endothelial dysfunction, and increased cell death^57^. Skeletal-muscle atrophy is pronounced due to decreased loading and changes in circulating levels of endocrine hormones and steroids^58^, while bone loss is also a critical issue^59^. To counteract these effects, a host of advanced tissue-regeneration, bioprinting, and smart-drug-delivery technologies may be implemented^60^.

Fluid shift due to changes in circulation, altered bioavailability, and metabolic changes in enzyme activity can alter the pharmacokinetics and dynamics of anaesthetic drugs in space^61^. Results achieved by Earth-based ‘head down bed rest’ studies simulating a response to microgravity have determined that propofol is a suitable general anaesthetic, with simulated microgravity only having a minor impact on drug pharmacokinetics^61^; lidocaine efficacy has similarly shown equivalence between controls and simulated microgravity^61^, while studies have listed ketamine as the ideal intravenous anaesthetic for use in space^62^. Regional anaesthesia requires fewer resources, permits quicker recovery of the patient, and does not require laryngoscopy and intubation, but has a longer learning curve compared with general anaesthesia and is not suited to more complex, deep laparoscopic, or major open surgery^63^. General anaesthesia is simpler and intubation and laryngoscopy have previously been demonstrated as feasible in microgravity^64^. Ensuring the right conditions for storage and shielding from radiation will be required to preserve the shelf life of drugs on board. Intravenous bags and lines will have to be degassed before launch, as air bubbles behave differently in microgravity and can be introduced into the patient’s circulation as life-threatening air emboli^30^.

## Features of an ideal space surgical robotic set-up

### Hardware features

Given the SWaP constraints on spacecraft, an ideal surgical robot must be light, compact, and power efficient, and provide multifunctional capabilities beyond surgery^44^. It should routinely perform non-surgical tasks, for instance handling and manipulation of sensitive materials for scientific experiments, and convert to a surgical robot using hardware adapters or exchangeable end effectors/instruments when a situation arises^40,44^. The system should support a wide range of instruments, including ultrasonographic probes, suction and irrigation lines, and advanced energy. Instruments and arms should be able to function within a surgical containment unit.

Single-port devices align with the requirements of space surgery. They occupy less volume and require fewer components. In neutral configuration, the instruments occupy a compact, linear profile, which may allow for easier mating with a containment chamber. As the instruments triangulate within a more compact working area, by-products such as diathermy smoke are concentrated in a smaller volume. This creates the potential for integrated, fixed suction through channels built into the instruments or endoscope.

Durability and reliability of components is paramount, as resupply will be near impossible during long-term extraplanetary missions. Extensive preflight stress testing will be required to ensure the system will withstand g overload and vibration during launch and radiation during spaceflight^65–67^. Motor drivers, servos, and microcontrollers will need to be protected from radiation, possibly through aluminium/titanium shielding. Ideally, structural components should be 3D printable on board^44^. The system should feature a modular design, allowing for the rapid replacement of defective complex components, with prebuilt replaceable units, as opposed to extensive manual repair. This principle is reflected on board the ISS, with several key components for communication, electrical control, storage, and life support built as swap-in swap-out orbital replaceable units. Materials used will have to adhere to NASA off-gassing, electromagnetic interference, corrosion resistance, and flammability requirements for habitable volumes^68^. Surgical-grade materials for patient-contact components (titanium, PEEK, and medical-grade polymers) are typically inherently inert and have very low off-gassing rates. The greater challenge lies in housing, insulation, adhesive, and coating materials that are all potential sources of off-gassing.

Effective thermal management is a key engineering challenge in microgravity. Although convection-based cooling is provided artificially, buoyancy-driven convection does not take place^69^. Heat generated by actuators within robotic systems and during diathermy will not dissipate quickly, increasing the risk of unintended tissue damage, hardware malfunctions, and operator injury. Warmed CO_2_ insufflation gas is routinely used in surgery to prevent hypothermia, yet, in space, this may result in hyperthermia. To address this, conduction paths, touch-temperature coating, and miniature fans should be included in instruments and robotic components^68^ . Alternative strategies for achieving haemostasis should be explored, including pulsed cutting, advanced energy, laser, fast-acting fibrin gels, and clips^70^. Robotic actuators should use brushless direct-current motors due to compactness, a high torque-to-weight ratio, efficient heat dissipation, and lack of brush dust^71^.

### Software, human–robot interaction, and autonomy

Robotic assistance will likely take the form of artificial intelligence (AI)-based real-time decision-making support, embedded internally within the master console, as well as externally through interactive humanoid and free-flying robots^2,44^. On the master-console interface, this assistance would manifest as augmented-reality overlays of key structures, flagging of critical events requiring operator intervention (for example highlighting bleeding to be cauterized), haptic cues to ‘nudge’ the operator towards the target, indications for instrument exchange, and context-aware suggestions for the next set of actions in the procedure^72,73^.

**Fig. 3.**
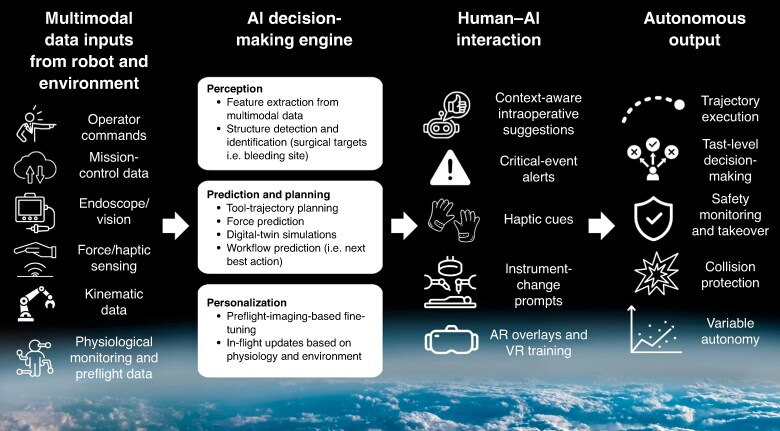
Overview of how multimodal inputs are used by an on-board AI decision engine to enable perception, prediction, personalization, and autonomous action during space surgery AI, artificial intelligence; AR, augmented reality; VR, virtual reality.

AI models performing decision-making tasks should use multimodal data (including vision, kinematic data, haptics, vitals, notes, and crew commands) (*Fig. 3*). These models should be extensively pretrained on Earth and personalized using astronaut-specific preflight data (including imaging, physiological parameters, and medical history) to enable individualized surgical support. During the mission, weights should be continuously adapted to reflect physiological changes over time^74^. Although real-time telesurgery is impossible, potential AI solutions have been proposed to bridge the gap between surgeon and robot, including bandwidth optimization, simulated surgery on digital twins to extract tool-manipulation data for use in high-level control policy, and haptic/kinematic forecasting^75^.

With regards to autonomy, robots must be capable of variable function, ranging from level one (basic tremor filtration and motion scaling) to level four (supervised autonomy)^44,52^. A local autonomy approach can be implemented—the operator provides high-level information (for instance suture points X and Y) and the robot generates a trajectory, presents the plan to the operator to accept or modify, and then executes the task without human input. In practice, this will likely not rely on a single, generalized algorithm, but on specialized agentic AI models that adapt under uncertainty, collaborate across roles towards a unified goal, and interface with operators on the spacecraft and on Earth.

Telementoring has been shown to effectively train novice operators to perform a range of surgical procedures in microgravity. Astronauts should receive ongoing training throughout their mission (not only during emergency scenarios) to build new skills and maintain proficiency in existing ones^44^. As much as possible, the surgical robot operator interface should mirror the design of control systems already used on board. For instance, large external robot arms such as the Canadaarm2 and Dextre are operated via two joysticks for rotational and translational control. While the scale and precision are very different, adopting a similar control strategy would shorten the learning curve for the surgical robot. VR could play an important role in training and preoperative simulation, allowing the surgeon to familiarize themselves with patient-specific anatomy based on preflight MRI scans^44^. Humanoid robots analogous to the Robonaut should collect and analyse telementoring video from Earth-bound experts to generate simulation drills, assessing performance and providing corrective feedback. During surgery, such robots should have the capability to communicate with the surgeon, answer queries, ensure that preoperative preparation follows standard checklists, and provide basic physical assistance in instrument exchange and handling of disposables.

### Cloud computing and network infrastructure

**Fig. 4.**
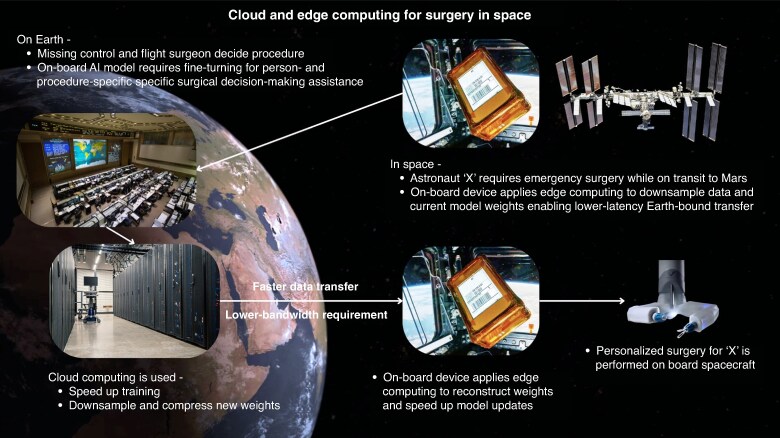
Cloud–edge-computing-supported pipeline for AI model updates during space surgery AI, artificial intelligence.

In a space context, cloud computing can be understood as a two-tier system: Earth-based and spacecraft-based edge computing units. On Earth, conventional cloud computing can perform intensive tasks such as model training, 3D reconstructions, simulation, image processing, and robot kinematics and control. Instead of transmitting raw data, the cloud can be used to compress and downsample inputs into high-level, low-bandwidth information, reducing latency when transmitted using existing communication frameworks like the TDRSS. On board a spacecraft, edge computing can extend the cloud by applying enabling techniques such as virtualization and containerization, orchestration, and fault isolation. Such mechanisms allow limited computing hardware to support multiple functions such as robot control, model execution, and video processing in a more efficient manner (*Fig. 4*).

**Fig. 5.**
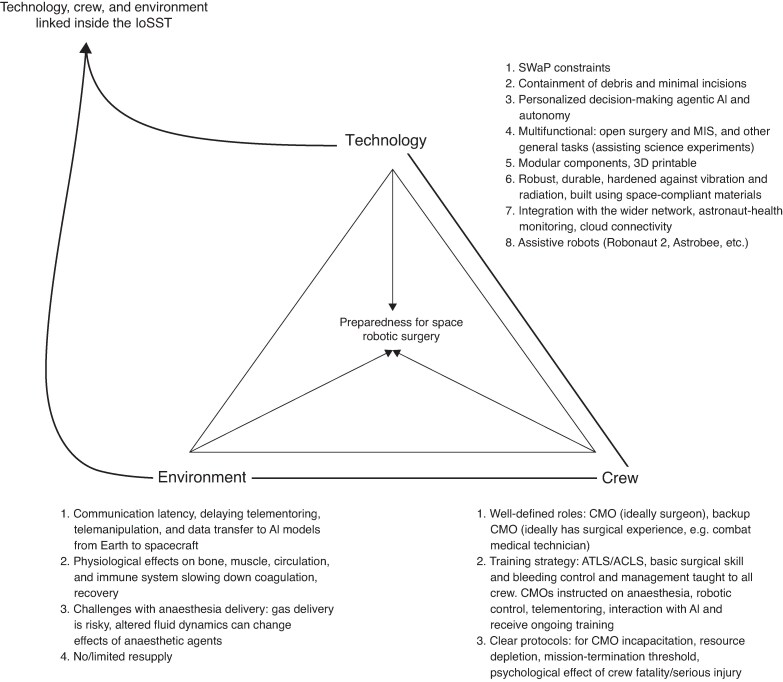
Contributing features that lead to a capable set-up for robotic surgery in space Crew, environment, and technology are linked to each other in the IoSST. IoSST, Internet of Space Surgical Things; SWaP, size, weight, and power; MIS, minimally invasive surgery; AI, artificial intelligence; 3D, three-dimensional; CMO, crew medical officer; ATLS, advanced trauma life support; ACLS, advanced cardiac life support.

Beyond the technical infrastructure of cloud and edge computing, a broader framework can be envisioned as an Internet of Space Surgical Things (IoSST) (*Fig. 5*). The IoSST interconnects all digital aspects of crew, environment, and robotic operation, enabling continuous data exchange between ground-based sources, crew-health monitors, environmental sensors, decision-making AI models, and the surgical robot. This creates integrated situational awareness and coordinated responses to a surgical event.

## Summary

Evidence-based prediction of medical events in space points to the inevitability of a surgical emergency on an extended-duration exploration mission. This necessitates a shift from current practice in space-based care, from ‘stabilize-evacuate’ to ‘stabilize-treat-recover-return to duties’. Open and minimally invasive techniques have been proven feasible in microgravity, as long as the operator and patient are restrained and an appropriate containment strategy is used. Robotic surgery is perhaps the optimal technique for space surgery, primarily as it can incorporate a degree of autonomy in the physical execution of tasks and the cognitive decision-making process while bringing the same Earth-based benefits of better dexterity, workspace, and visualization associated with robotic MIS. However, there are several technical and non-technical challenges to implementation of space surgery, including size and power constraints, crew training, the lack of teleoperability due to communication latency, the difficulty of providing anaesthesia in space, and slower recovery of patients due to changes to human physiology. Based on the operative context of such a system, the ideal surgical robot would be compact in size, multiuse, intuitive and adaptable in handling and set-up, and reliable, with extensive fail-safes, redundancies, and 3D-printable components. It should be optimized for efficient human–machine interaction and augment the operator through multimodal assistance and real-time decision-making support. The intersection of robot actuation and sensor technology, advanced imaging, 3D printing, and robotic autonomy, coupled with efficient communication, data transfer and crew training, will form the basis for safe and effective surgical care in space.

## Data Availability

Not applicable.
